# ASSOCIATION OF RAPID EYE MOVEMENT SLEEP AND APOE PROTEOTYPE TO COGNITIVE PERFORMANCE IN OLDER ADULTS

**DOI:** 10.1093/geroni/igad104.3195

**Published:** 2023-12-21

**Authors:** Edward Hammel, Mitchell Roberts, Erica Sappington, Paul Dagum, Jeffrey Iliff, Swati Rane Levendovszky, Jeff Lowenkron, Carla VandeWeerd

**Affiliations:** UF Precision Health Research Center - The Villages, The Villages, Florida, United States; UF Precision Health Research Center - The Villages, The Villages, Florida, United States; UF Precision Health Research Center - The Villages, The Villages, Florida, United States; Applied Cognition Inc, Redwood City, California, United States; University of Washington, Seattle, Washington, United States; University of Washington Medical Center, Seattle, Washington, United States; The Villages Health, The Villages, Florida, United States; UF Precision Health Research Center - The Villages, The Villages, Florida, United States

## Abstract

Exploring the multifaceted interaction of sleep, disease-related biomarkers and cognitive decline is of particular importance for aging populations. Sleep quality and the APOE E3/E4 allele has been linked to Alzheimer’s Disease (AD). This study aims to examine relationships between older adults’ level of cognitive performance, proportion of rapid eye movement (REM) sleep, and APOE proteotype. Analysis of sleep study data collected in an active lifestyle retirement community - The Villages, Florida was conducted using a subset of markers collected as part of a larger cognitive health study. Polysomnography, cognitive battery (Montreal Cognitive Assessment(MoCA), Trail Making Tests(TMT), Symbol Digit Modalities Test(SDMT), Psychomotor Vigilance Test(PVT)), and APOE blood-biomarker (E2/E3,E3/E3,E3/E4) data were obtained from healthy participants (n=33) aged 55-65. Descriptive statistics, multilinear regression, and ANOVA were used. Participants’ mean age was 61.9 years, with 55% being male. Cognitive test scores did not show a significant relationship with REM sleep percentage (p=0.3096). No consistent dispersion or skew was shown in box plot analysis of APOE proteotype across cognitive performance measures. Unexpectedly, although not statistically significant (p=0.396), average REM sleep percentage was higher in the APOE E3/E4 group (20.04%, Mean:75.44 minutes) as compared to the E3/E3 group (15.99%,Mean: 59.07 minutes). Proteotype E3/E4 was examined specifically due to its potential association with Alzheimer’s disease. The REM sleep proportion was lower than the average experienced on a typical night at home, possibly due to study conditions. Further research is needed examining the relationship between older adults with REM sleep insufficiency and cognitive impairment.

